# Self-Cleaning Cotton Obtained after Grafting Thermoresponsive Poly(*N*-vinylcaprolactam) through Surface-Initiated Atom Transfer Radical Polymerization

**DOI:** 10.3390/polym12122920

**Published:** 2020-12-05

**Authors:** Bhaskarchand Gautam, Hsiao-hua Yu

**Affiliations:** 1 Smart Organic Material Laboratory, Institute of Chemistry, Academia Sinica, Nankang, 128 Academia Road, Sec. 2, Taipei 115, Taiwan; gtmbhaskar@gmail.com; 2 Taiwan International Graduate Program (TIGP), Sustainable Chemical Science and Technology (SCST), Academia Sinica, Taipei 115, Taiwan; 3 Department of Applied Chemistry, National Chiao Tung University, Hsinchu 300, Taiwan

**Keywords:** smart textile, self-cleaning, poly(*N*-vinylcaprolactam), surface-initiated atomic transfer radical polymerization (SI-ATRP)

## Abstract

Although the performance of smart textiles would be enhanced if they could display self-cleaning ability toward various kinds of contamination, the procedures that have been used previously to impart the self-cleaning potential to these functional fabrics (solvent casting, dip coating, spin coating, surface crosslinking) have typically been expensive and/or limited by uncontrollable polymer thicknesses and morphologies. In this paper, we demonstrate the use of atomic transfer radical polymerization for the surface-initiated grafting of poly(*N*-vinylcaprolactam), a thermoresponsive polymer, onto cotton. We confirmed the thermoresponsiveness and reusability of the resulting fabric through water contact angle measurements and various surface characterization techniques (scanning electron microscopy, atomic force microscopy, Fourier transform infrared spectroscopy). Finally, we validated the self-cleaning performance of the fabric by washing away an immobilized fluorescent protein in deionized water under thermal stimulus. Fluorescence micrographs revealed that, after the fifth wash cycle, the fabric surface had undergone efficient self-cleaning of the stain, making it an effective self-cleaning material. This approach appears to have potential for application in the fields of smart textiles, responsive substrates, and functional fabrics.

## 1. Introduction

Self-cleaning technologies have inspired researchers since the 20th century [1]. They have been used to produce various materials, including textiles [2,3], window glasses [4,5], car mirrors [6,7], solar panels [8,9], and furnishing materials [10], possessing self-cleaning surfaces. Among these self-cleaning substrates, fabric surfaces are most significant because of their unique features and vast applications in the fields of smart textiles and self-disinfecting and contamination-free surfaces [11,12].

Compounds displaying external responsive behavior (e.g., thermal, pH responses, mechanical energy, etc.) have been used to generate smart fiber [13,14]. The most common have been thermoresponsive materials. Poly(*N*-isopropylacrylamide) (PNIPAAm) and poly(*N*-vinylcaprolactam) (PNVCL) have been the most frequently used polymers for grafting onto material surfaces due to their nonionic character, high solubility in water and organic solvents, and excellent absorption ability [15,16]. These two polymers display almost the same lower critical solution temperature (LCST) in water, between 30 °C and 32 °C [17]. Furthermore, the LCST is completely reversible for both polymers, ensuring that they undergo consistent swelling-to-collapsing transitions in aqueous solutions in the hydrogel state [18]. Although both materials have similar LCSTs, they display different thermodynamic properties and undergo different mechanisms of phase transition. PNIPAAm exhibits a thermoresponsive phase nature (type II), whereas PNVCL exhibits “classical” Flory–Huggins thermoresponsive behavior in water (type I), meaning that the cloud points of PNVCL-coated materials can be altered by controlling the polymer length, without using another comonomer, during the surface coating process [19]. In addition, transparency and high water solubility make PNVCL polymers desirable materials that are easy to fabricate upon various substrates [20,21,22].

Several grafting methods have been used to coat thermoresponsive polymers onto surfaces, including those based on radiation, dipping, cross-linking [23,24], electron-beam crosslinking polymerization [25], plasma polymerization or plasma deposition [26], solvent casting [27], spin coating and spray coating [28], and surface-initiated atom transfer radical polymerization (SI-ATRP) [29,30]. With the exception of SI-ATRP, all of these techniques either lack flexibility or are expensive when one attempts to control the polymer thickness or morphology [29].

SI-ATRP is a promising technique for coating thermoresponsive polymers onto fabric surfaces because it allows control over not only the polymer film thickness but also the polymer surface morphology [31]. Using the SI-ATRP concept, Karesoja et al. [32] grafted poly(ethylene oxide–*block*–PNVCL) onto mesoporous silica particles, then attached a poly(ethylene glycol) block through click chemistry; the resulting grafted silicon particles could disperse well in water at 35 °C, and could also lose their hydration layer at temperatures above 37 °C. Similarly, in 2013, Kavitha et al. [33] reported the use of in situ ATRP grafting of *N*-vinylcaprolactam (NVCL) onto graphene oxide (GO) to generate GO-*g*-PNVCL materials that displayed thermal aggregation behavior at temperatures close to 38 °C.

**Figure 1:**
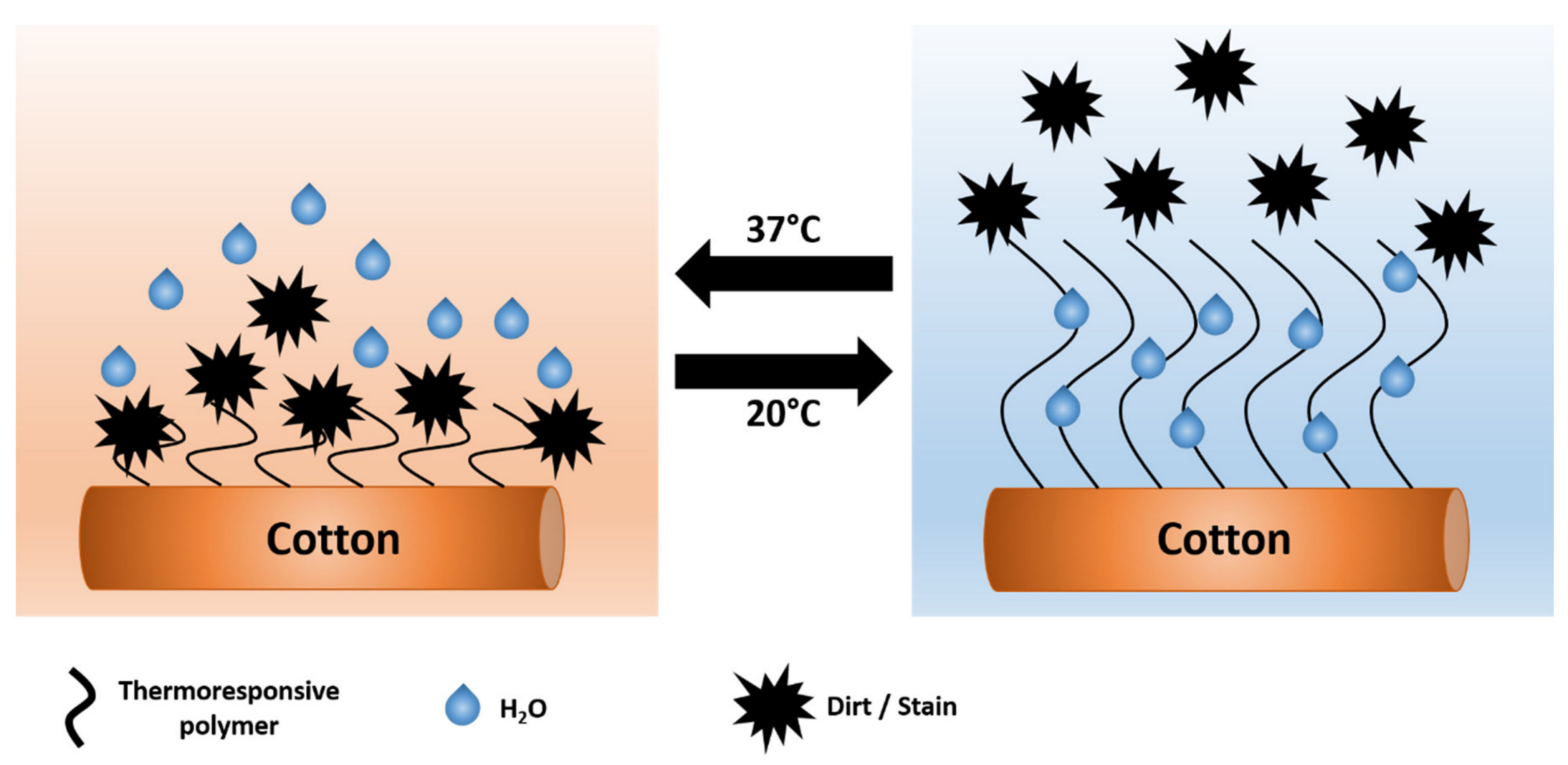
Schematic representation of the self-cleaning of a thermoresponsive polymer-grafted cotton fabric.

Although several attempts have been made to graft PNIPAAm using SI-ATRP [33,34,35,36], grafting of PNVCL onto cotton fabric through SI-ATRP has been relatively unexplored. For example, Crespy et al. [37] used methacrylic acid as a comonomer to graft PNVCL through SI-ATRP onto a cotton textile surface to produce a temperature- and pH-responsive fabric. In this present paper, we demonstrate the first example of the successful grafting of thermoresponsive PNVCL onto 100% cotton fabric through SI-ATRP, as illustrated in Figure 1. We evaluated the grafting efficiency by calculating the time-dependent grafting yield. We verified the successful grafting of PNVCL by using Fourier transform infrared (FTIR) spectroscopy, scanning electron microscopy (SEM), atomic force microscopy (AFM), and water contact angle (WCA) measurements. To confirm the thermoresponsive nature of the cotton fabric, we used WCA analysis to measure the changes in the hydrophobicity of the cotton fabric surface upon thermal stimulation. Similarly, we verified the recyclability of the newly polymer-grafted fabric surface over several wash cycles. Moreover, to highlight its practical applicability as a self-cleaning material, we demonstrate that an immobilized protein could be washed away from this modified fabric only under thermal stimulus. We suspect that such responsive fabrics would have many applications in smart textiles, in the health industry, and as building materials.

## 2. Materials and Methods

### 2.1. Materials

3-Aminopropyltriethoxysilane (APTMS, 98%), α-bromoisobutyryl bromide (BiBB, 98%), copper bromide (CuBr, 99%), *N*,*N*,*N*″,*N*″,*N*″-pentamethyldiethylenetriamine (PMDETA, 99%), triethylamine (TEA), dichloromethane (DCM, 99.5%), methanol (MeOH), and toluene were purchased from Sigma–Aldrich (St.Louis, MO, USA). Cotton fabrics were obtained from a local fabric store (100% plain cotton, 60 × 60 thread count). Cy5-streptavidin was purchased from Thermo Fisher Scientific (Cat. No. 434316, Camarillo, CA, USA); *Aequorea victoria* green fluorescent protein (GFP) was obtained from Sini Biological (Cat. No. 69001-S08E, St. BPA, Beijing, P.R. China). Prior to treatment, the cotton fabrics were washed sequentially with ethanol, distilled water, and acetone through ultra-sonication, each time for 15 min to remove any impurities.

### 2.2. SI-ATRP Initiator Immobilization on Cotton Fabric

A cleaned cotton fabric was immersed in a reactor containing toluene (200 mL) and APTMS (1 mL) and stirred for 1 h at room temperature. The fabric was then washed with MeOH and distilled water (three times each) to remove any non-bonded APTMS. Next, the fabric was immersed in DCM (200 mL) containing TEA (10 mL) and cooled in an ice bath for 30 min. BiBB (10 mL, 81 mmol) was added dropwise to the cold solution, which was then stirred overnight at room temperature. Finally, the fabric was washed with DCM and distilled water (three times each).

### 2.3. Fabrication of PNVCL-Grafted Cotton Fabric through SI-ATRP

CuBr (130 mg, 0.90 mmol) and PMDETA (0.25 g, 1.5 mmol) were added to the initiator (BiBB)-coated cotton fabric immersed in a mixture of MeOH and H_2_O (1:1). The monomer NVCL (3.2 g, 23 mmol) was added, and then the mixture was stirred for 12 h under a N_2_ atmosphere. Finally, the fabric was washed with MeOH and distilled water (three times each) and vacuum-dried overnight.

### 2.4. Surface Characterization

SEM with energy-dispersive spectroscopy (SEM/EDS; Zeiss Ultra Plus, Oberkochen, Germany) was used to examine the fabric surface morphology and elemental composition before and after polymer grafting. FTIR spectroscopy (PerkinElmer Spectrum, Waltham, MA, USA) was used to monitor the covalent bonding of the initiator and the polymer onto the cotton surface. WCA measurements (Digidrop contact angle meter, Dublin, Ireland) were conducted using a sessile drop method with droplets of deionized water (10 µL). AFM, Veeco di Innova (Billerica, MA, USA) was employed in tapping mode to investigate the surface roughness and morphology of the modified cotton fabric surface. Fluorescence images of Cy5-streptavidin and GFP immobilized on fabric surfaces were recorded using a Nikon-Eclipse fluorescence microscope (Winooski, VT, USA).

### 2.5. Thermoresponsive Behavior of Polymer-Grafted Cotton Fabrics

To investigate the thermoresponsiveness of the polymer-grafted fabric, WCAs were recorded using droplets of deionized water (10 µL). The drop was placed gently onto the surface of the fabric heated at 37 °C, and its WCA was measured. The same fabric was cooled to 20 °C, and then the WCA was recorded again. This cycle of WCA measurements was repeated four more times to confirm the transition of the hydrophobic and hydrophilic properties of the polymer-grafted fabric surface.

### 2.6. Self-Cleaning PNVCL-Grafted Cotton Fabric

Cy5-streptavidin (100-fold diluted in deionized water) and GFP (0.25 mg/mL) were used to evaluate the self-cleaning potential of the thermoresponsive fabric. A sample (1.5 cm × 1.5 cm) of the modified fabric was incubated in a solution of Cy5-streptavidin or GFP for 1 h at room temperature to allow the protein to attach onto the surface. The polymer-modified fabric (and a corresponding control fabric) was then washed by dipping five times into deionized water at 20 °C; fluorescence images were recorded (with excitation/emission at 650/670 nm for Cy5-streptavidin; 487/508 nm for GFP). These washing cycles were repeated five more times to ensure that the protein had been washed away from the surface. To quantify the thermoresponsiveness of the modified fabric, washing at 37 °C was also performed, and the fluorescence intensity was compared with that of the control. The same experimental procedures were applied for the fabric without the grafted polymer, used as a control in this study. The fluorescence intensity was calculated using ImageJ software; the auto-fluorescence intensity of the pure cotton fabric was subtracted each time prior to plotting the fluorescence intensity graph.

## 3. Results and Discussion

### 3.1. Grafting the Thermoresponsive Polymer onto Cotton Fabric through SI-ATRP

**Scheme 1:**
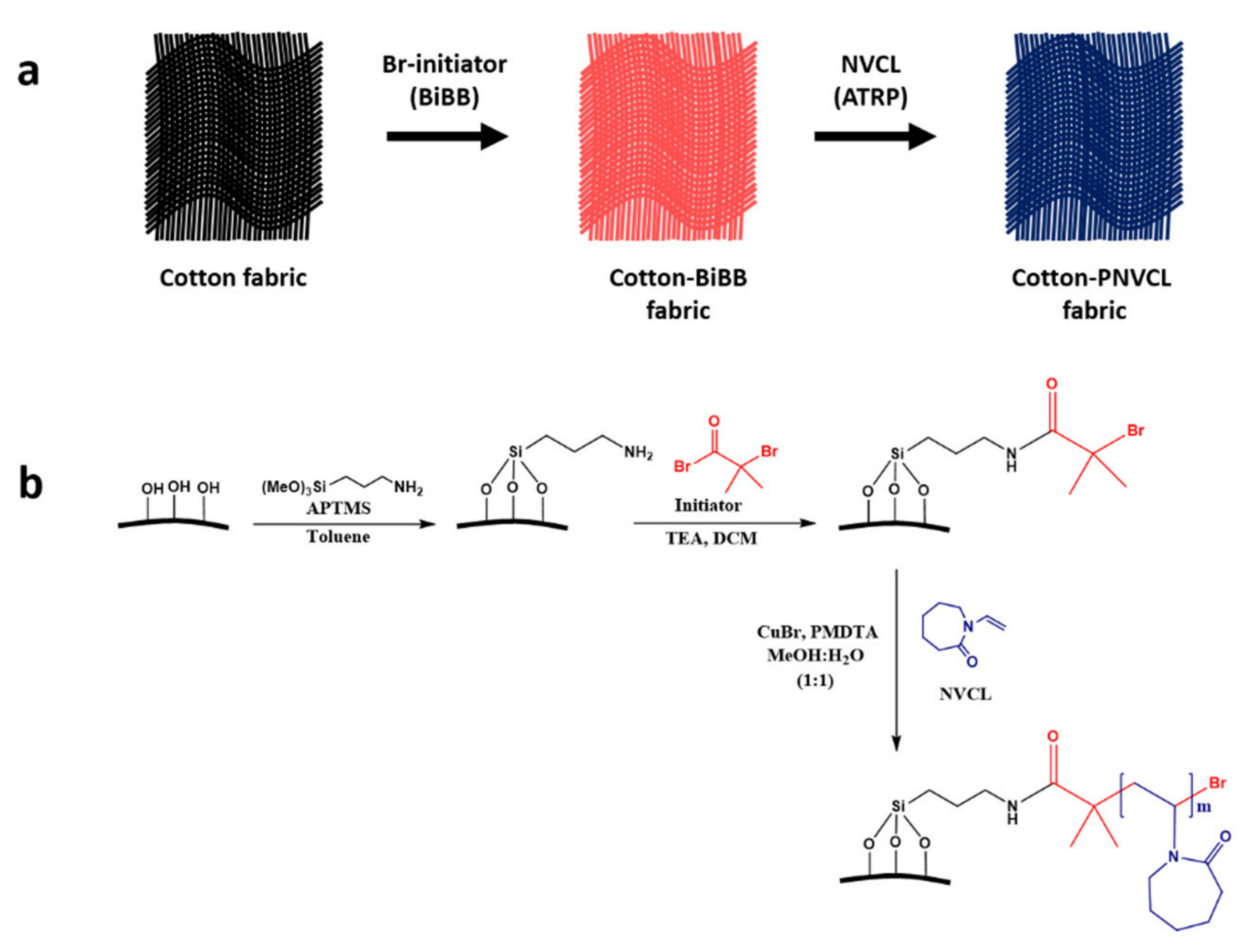
(**a**) Schematic representation of the grafting of poly(*N*-vinylcaprolactam) (PNVCL) onto cotton through surface-initiated atomic transfer radical polymerization (SI-ATRP). (**b**) Chemical reactions involved in each step of polymer grafting onto the cotton surface.

The procedure for grafting the thermoresponsive PNVCL involved two steps: (i) immobilization of the ATRP initiator (BiBB) to produce a BiBB-coated cotton fabric and (ii) radical polymerization of NVCL to produce the PNVCL-grafted cotton fabric (Scheme 1a). The chemical reactions in Scheme 1b reveal that OH groups of the cellulose were linked initially with the Si atom of the APTMS linker to generate an amino-functionalized cotton fabric surface. The ATRP initiator BiBB was then conjugated to this surface under basic conditions to form a bromo-initiator-modified cotton fabric (cotton-BiBB). In the presence of CuBr as a catalyst, cotton-BiBB generated free radicals to initiate polymerization of the C=C groups of units of the monomer NVCL, thereby generating long chains of compact PNCVL on the cotton surface (cotton-PNCVL). The progress of the grafting was monitored in terms of grafting yield analysis; the successful grafting of the polymer onto the cotton surface was confirmed through WCA, FTIR spectral, SEM, and AFM analyses.

Traditional polymer coating methods such as dip coating and spin coating pose various challenges such as difficulty in controlling material thickness onto the surface. Furthermore, these techniques mainly rely on physical interactions with the substrate, which often result in easy detachment. In contrast, the SI-ATRP-based approach reported here to make a polymer film provides easy control over film thickness via strong covalent interactions.

### 3.2. Polymerization Kinetics

We used grafting yield analysis to evaluate the grafting efficiency of PNCVL onto the cotton fabric. The grafting was monitored in terms of the weight increase of the fabric with respect to the polymerization time. The grafting yield (%) is defined as

Grafting yield (%) = (*W*_b_ − *W*_a_)/*W*_a_ × 100 (1)

where *W*_a_ and *W*_b_ represent the weights of the dried cotton fabric before and after polymerization, respectively, at various polymerization times.

**Figure 2:**
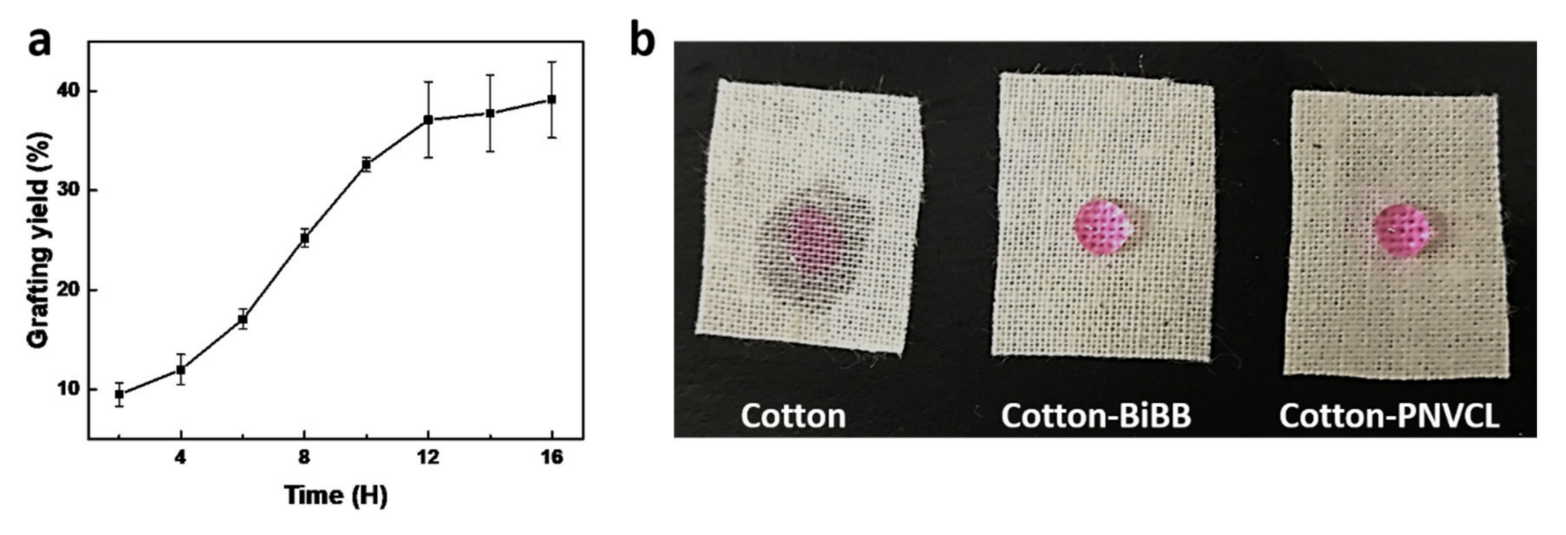
(**a**) Grafting yield of PNVCL grafted onto pure cotton, plotted with respect to the polymerization reaction time. (**b**) Photograph of samples of cotton, cotton-α-bromoisobutyryl bromide (cotton-BiBB), and cotton-PNVCL fabrics treated with a water drop, revealing the differences in hydrophobicity before and after polymer grafting.

We performed the polymerizations for various lengths of time (from 2 to 16 h) and calculated the grafting yield in each case (Figure S3). The curve in Figure 2a reveals that the grafting yield increased relatively linearly from 9.5 to 37.1% upon increasing the polymerization time from 2 to 12 h, but it remained almost constant thereafter (37.1, 37.8, and 39.2% after 12, 14, and 16 h, respectively); hence, we fixed the polymerization time at 12 h for subsequent experiments. To confirm the successful modification of the cotton surface after 12 h of polymerization, we placed droplets of rhodamine-B dye-stained deionized water (10 µL) gently onto the surfaces of the unmodified cotton fabric (cotton) and the cotton-BiBB and cotton-PNVCL fabrics, then compared their wettability. As expected, at room temperature, cotton displayed superhydrophilic behavior, with complete absorption of the water drop (Figure 2b). In contrast, both cotton-BiBB and cotton-PNVCL displayed hydrophobicity (nonwettability), as evidenced by water-repellent characteristics (Figure 2b), in full agreement with the results of a previously published study [30].

### 3.3. Surface Characterization of Thermoresponsive Polymer-Grafted Fabric

**Figure 3:**
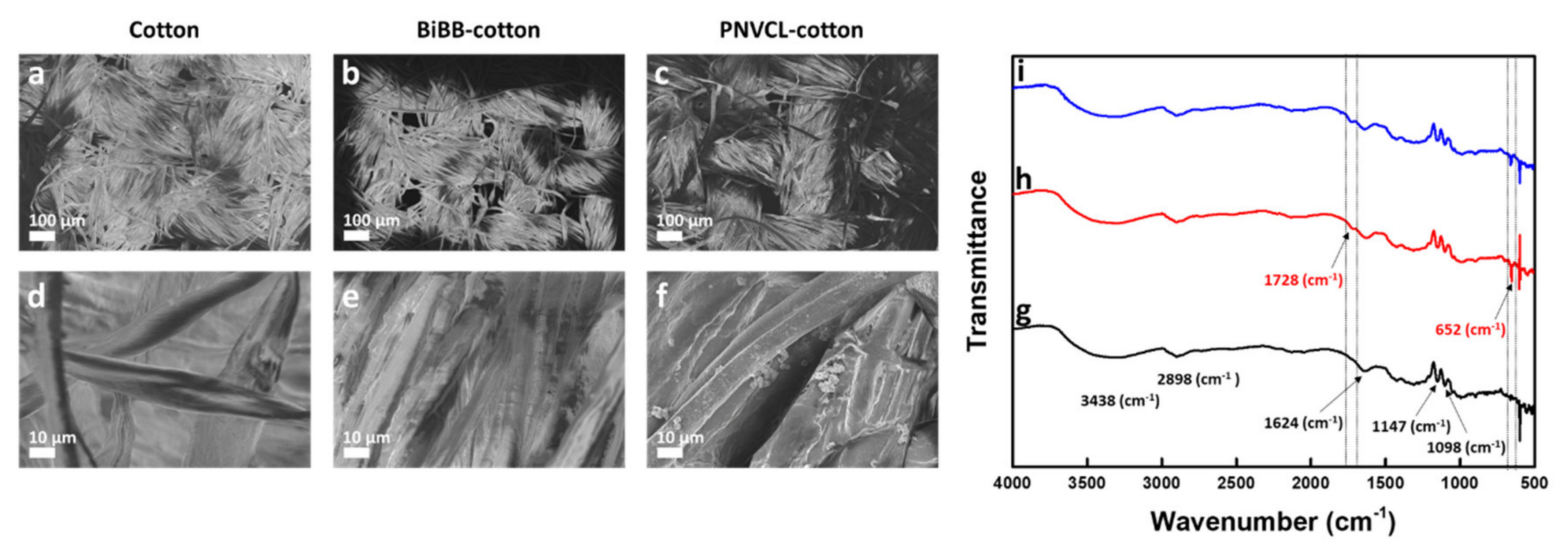
(**a**–**f**) SEM images of the (**a**,**d**) cotton, (**b**,**e**) cotton-BiBB, and (**c**,**f**) cotton-PNVCL fabrics; top images magnified 100×; bottom images magnified 1000×. (**g**–**i**) Fourier transform infrared (FTIR) spectra of the (**g**) cotton, (**h**) cotton-BiBB, and (**i**) cotton-PNVC fabrics.

We used SEM and AFM to examine the morphologies and roughness of the cotton surfaces. SEM revealed that cotton had a smooth surface, whereas cotton-BiBB and cotton-PNVCL had rougher surfaces after we coated the initiator and polymer, respectively, onto the fabric surface (Figure 3a–f). SEM/EDS quantification (Figure S1) revealed increments in the content of Br atoms of 1.3 wt % for the initiator-coated fabric and 0.81 wt % for the polymer-grafted fabric; as expected, no signal for Br atoms appeared in the EDS spectrum of the pure cotton. The findings are consistent with the successful grafting of PNVCL onto the fabric. The roughness of the fabric surfaces was further examined using 3D AFM and quantified by measuring their root-mean-square (RMS) roughness (Figure S2). An almost two-fold increase in RMS (1.3 µm) occurred for cotton-BiBB relative to that of cotton (0.7 µm), consistent with efficient coating of the ATRP initiator. Gratifyingly, after grafting the polymer, the fabric surface exhibited a three-fold increase in its RMS roughness (2.1 µm) relative to that of cotton. Thus, both the AFM imaging and RMS data verified the effective SI-ATRP-mediated grafting of PNVCL onto the cotton fabric, supporting the SEM results and consistent with previous findings [38,39].

Figure 3g–i presents the FTIR spectra of the fabrics cotton, cotton-BiBB, and cotton-PNVCL, respectively. The strong peak observed in each spectrum at 3438 cm^−1^ is characteristic of the stretching vibrations of the OH groups of cellulose lignin and water [40]. The signal at 2898 cm^−1^ is due to the C–H stretching vibration of cellulose and hemicellulose [41]. The peak at 1624 cm^−1^ may be due to water present in the fabric [42]. The two peaks at 1147 cm^−1^ and 1098 cm^−1^ are due to C–O stretching in cellulose [43]. A characteristic peak for C=O stretching at 1728 cm^−1^ was evident only in the spectra of the BiBB- and PNVCL-grafted fabrics. Moreover, a minor increase in the transmittance intensity of the signal for the C=O groups was observed in the spectrum of the PNVCL-grafted fabric because of the extra C=O groups present in the caprolactam rings. Similarly, a signal for C–Br stretching appeared at 652 cm^−1^ in the spectra of cotton-BiBB and cotton-PNVCL; it was absent from the spectrum of cotton, consistent with the successful grafting of BiBB and PVCL in the former pair of fabric surfaces [43,44,45].

### 3.4. Surface Wettability of Initiator- and Polymer-Grafted Fabrics

**Figure 4:**
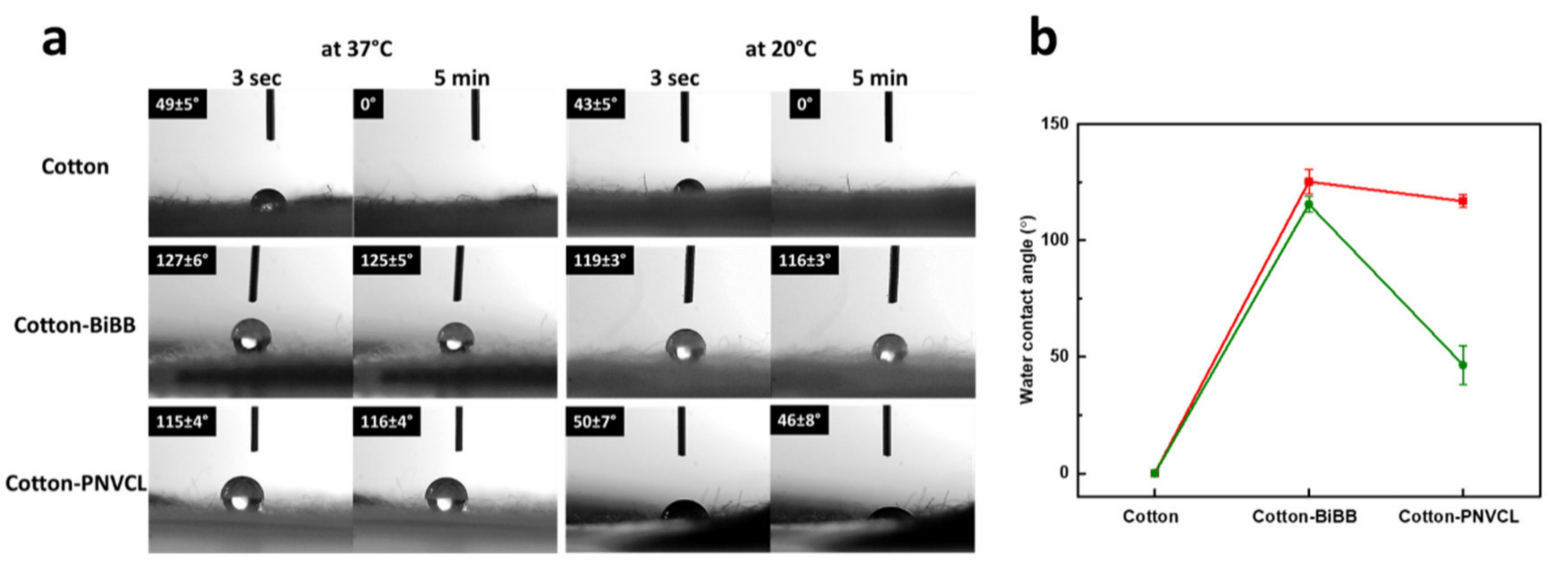
(**a**) Water contact angle (WCA) measurements at the surfaces of the cotton, cotton-BiBB, and cotton-PNVCL fabrics at 37 and 20 °C, with photographs taken after drop times of 3 s and 5 min. (**b**) Quantitative analysis of the WCAs at 37 °C (red) and 20 °C (green) after a drop time of 5 min.

We performed surface wettability analyses to examine the polymer grafting and the thermoresponsive nature of the modified cotton fabric. In this study, we determined the WCAs of cotton-BiBB and cotton-PNVCL through sessile-WCA measurements at 20 and 37 °C. Cotton was used as a control. Figure 4a displays WCA images of the three samples after drop times of 3 s and 5 min. Figure 4b plots the WCAs at 20 and 37 °C after a drop time of 5 min, providing a comparison of the surface wettability before and after polymer grafting. The pure cotton fabric displayed a WCA of 0° after 5 min at both 20 and 37 °C, consistent with the superhydrophilicity of cotton. After BiBB coating and polymer grafting, however, the WCAs increased dramatically to 125 and 116°, respectively, consistent with coating and modification of the cotton fabric surface. Importantly, upon changing the temperature from 37 to 20 °C, we did not observe any obvious change in the WCA for the BiBB-coated fabric, whereas the WCA for the polymer-grafted fabric decreased from 116 to 46°. This change in wettability for cotton-PNVCL at 20 °C was presumably due to the thermoresponsive nature of the grafted PNVCL.

Solid substrates coated with PNVCL using chemical deposition methods display nearly 70° change in WCA after thermal stimulus. In contrast, the smart fabric reported here displayed a 110° change upon application of thermal stimulus, highlighting superior thermoresponsiveness [46].

### 3.5. Reversibility of the Wettability Transition of the Thermoresponsive Polymer-Grafted Fabric

**Figure 5:**
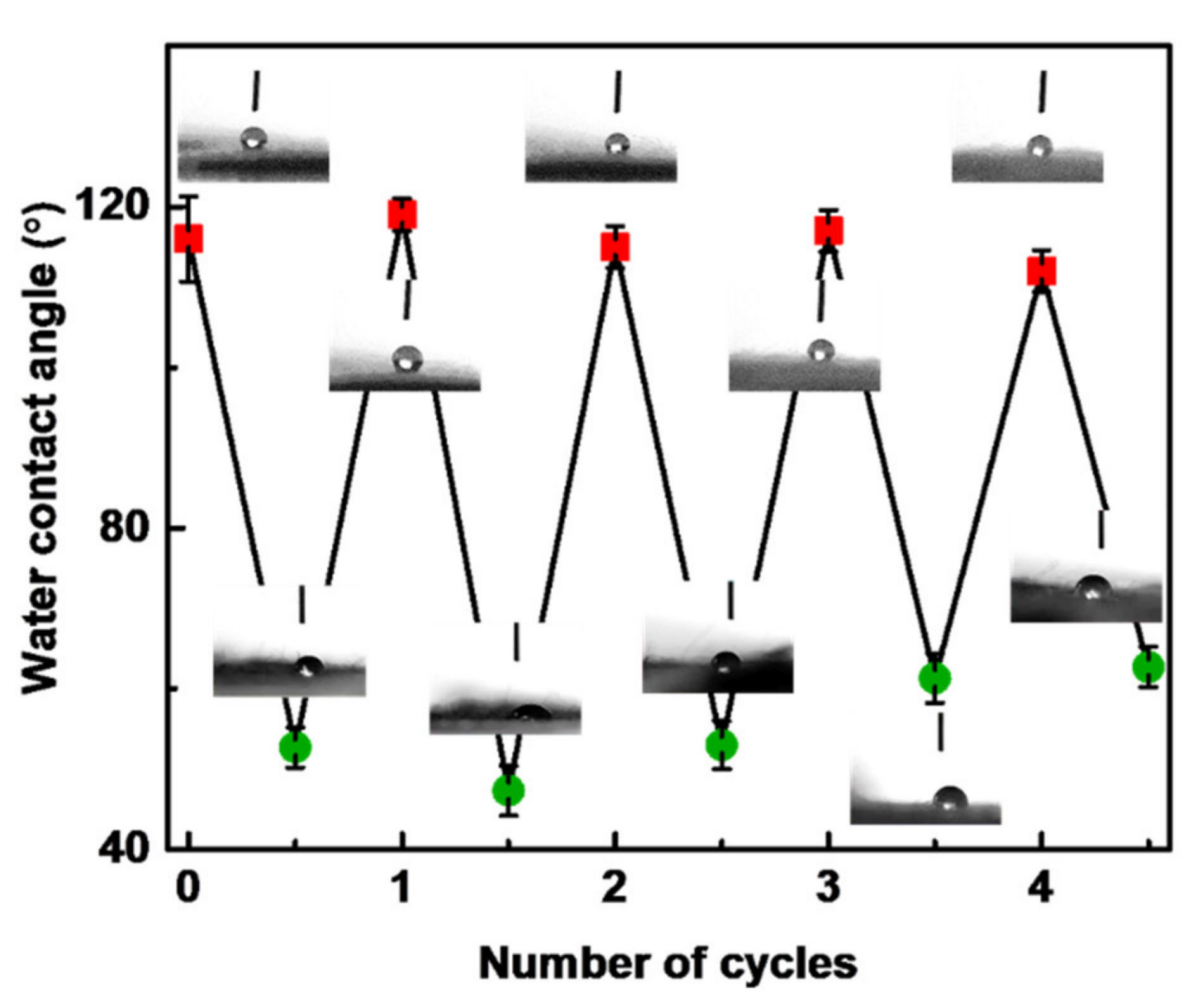
Reversibility of the WCA on the PNVCL-grafted cotton fabric after cycling the temperature between 37 and 20 °C. Insets: Photographs of the water droplets at 37 °C (red) and 20 °C (green).

After observing the thermoresponsive behavior of the modified fabric, we studied the reversibility of the switchable wetting of the modified cotton fabric. For this study, we measured WCAs initially after exposure of the fabric to a water droplet at 37 °C, then after the fabric had been cooled to 20 °C, and finally after the fabric had been heated to 37 °C; we consider this procedure to be a single wettability reversibility cycle. This procedure was repeated for five cycles. Figure 5 plots the WCAs with respect to the number of wettability reversibility cycles. In every thermal cycle, the wettability switched from hydrophobic at 37 °C (WCA: >100°) to hydrophilic at 20 °C (WCA: <55°); this process could be repeated for more than five cycles without substantial degradation of the thermoresponsive performance. Thus, even after five cycles of thermal stimulus, the morphology and thermoresponsive potential of the modified fabric remained intact. We suspected that this modified cotton fabric would not only possess self-cleaning properties but also display reusability for several wash cycles.

### 3.6. Self-Cleaning of the PNVCL-Grafted Cotton Fabric

**Figure 6:**
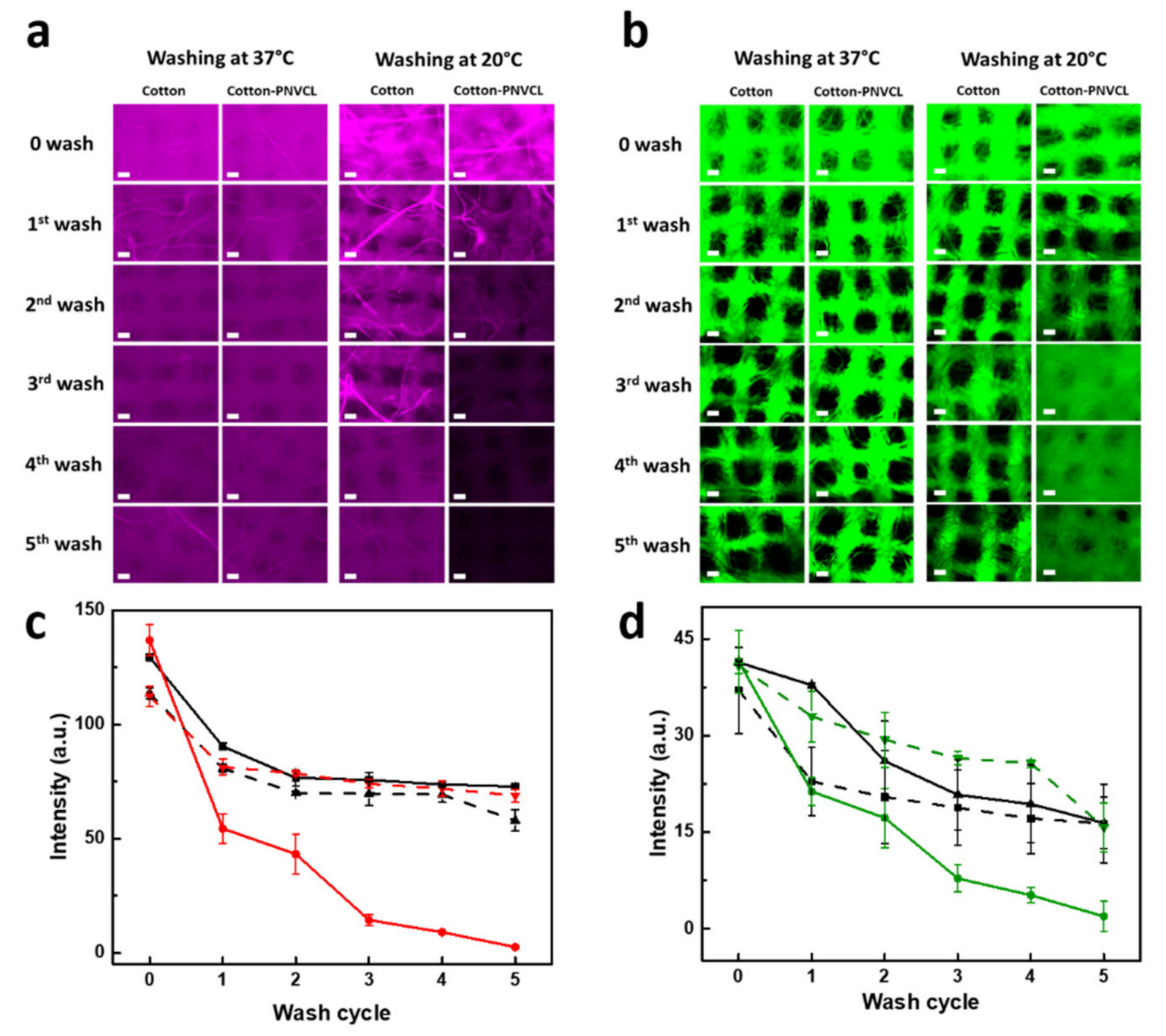
Fluorescence micrographs of (**a**) streptavidin-Cy5 and (**b**) green fluorescent protein (GFP) immobilized on cotton and cotton-PNVCL, recorded after washing 0, 1, 2, 3, 4, and 5 times at 37 and 20 °C; scale bars: 100 µm. (**c**,**d**) Fluorescence intensities of (**c**) streptavidin-Cy5 and (**d**) GFP on cotton (black lines) and cotton-PNVCL (red/green lines) plotted with respect to the number of wash cycles at 37 °C (dashed lines) and 20 °C (solid lines).

We used Cy5-streptavidin and Aequorea victoria GFP to evaluate the self-cleaning efficiency of our thermoresponsive fabric. After immobilization of the protein (Cy5-streptavidin or GFP) onto the surface of the responsive fabric through physical interaction, we gently washed the fabric five times in deionized water, separately at 37 and 20 °C. We recorded fluorescence images of the fabrics and quantified them after each washing cycle. Figure 6a,b displays the fluorescence micrographs recorded after each wash cycle of the fabrics featuring immobilized Cy5-streptavidin and GFP, respectively; Figure 6c,d presents their respective quantitative analyses. As expected, there were only slight decreases in the fluorescence intensities of the protein-immobilized cotton and cotton-PNCVL after each washing cycle at 37 °C (Figure 6a,c). Furthermore, at 20 °C, we did not observe any substantial changes in the fluorescence intensity of the cotton surface for either protein after five cycles of washing (Figure 6a–d). In contrast, when we washed cotton-PNCVL at 20 °C, the fluorescence intensities at the surfaces of cotton-PNCVL decreased almost linearly, from 136 to 2.5 a.u. for Cy5-streptavidin (Figure 6a,c) and from 41 to 2.5 a.u. for GFP (Figure 6b,d). In other words, the thermoresponsive polymer-modified surface was the only material that responded to the temperature change, leading to decreased immobilization and efficient removal of Cy5-streptavidin and GFP. Thus, the self-cleaning properties and wettability of cotton-PNCVL both depended on the transition from a hydrophobic to hydrophilic surface upon applying the thermal stimulus.

The existing technology focusing on fabrication of self-cleaning fabrics heavily relies on utilization of nanoparticle-based systems (e.g., TiO_2_, Ag/ZnO) [12] which tends to increase the overall cost as well as increase optimization steps for development of the fabric. In contrast, the SI-ATRP-based approach demonstrated here is simpler and more economical, which may make it an attractive candidate for design of smart fabrics.

## 4. Conclusions

In conclusion, we have fabricated a thermoresponsive textile via SI-ATRP-mediated grating of PNVCL onto cotton fabric. The grafting yield varied linearly with time. The smart fabric displayed hydrophilic behavior at temperatures below 20 °C and transitioned to a hydrophobic nature above 32 °C. The modified fabric displayed remarkable self-cleaning properties, suggesting its prospective applications in smart fabric. We also showed that nearly 96% of the conjugated protein can be removed from the fabric following five washes, highlighting its thermoresponsive smart effect. Additionally, this highly effective smart fabric can also be recycled as shown by remarkable contact angle measurement. This highly economical and eco-friendly smart fabric may exhibit potential applications in the textile industry and health sector due to its ability to undergo laundering with minimal time, resources, and energy consumption. Moreover, we suspect this fabric would also have applications in the fields of functional and smart textiles.

## Supplementary Materials

The following are available online at https://www.mdpi.com/2073-4360/12/12/2920/s1, Figure S1: EDS spectra including an elemental measurements of the Cotton, Cotton-BiBB and Cotton-PNVCL fabrics. Figure S2: AFM images and roughness quantification of Cotton, Cotton-BiBB and Cotton-PNVCL fabrics. Figure S3: Quantitative data for grafting yield.
