# Supplementary material for: Self-Cleaning Cotton Obtained after Grafting Thermoresponsive Poly(N-vinylcaprolactam) through Surface-Initiated Atom Transfer Radical Polymerization
Source: Polymers (Basel). 2020 Dec 5;12(12):2920. doi: 10.3390/polym12122920 (PMC7762131; doi:10.3390/polym12122920)
Supplement: Supplementary file 1 [file polymers-12-02920-s001.pdf]

polymers

MDPI

Article

Self-Cleaning Cotton Obtained After Grafting Thermoresponsive Poly(*N*-vinylcaprolactam) Through Surface-Initiated Atom Transfer Radical Polymerization

Bhaskarchand Gautam[1,2,3] and Hsiao-hua Yu[1,3,4*]

[1] Smart Organic Material Laboratory, Institute of Chemistry, Academia Sinica, Nankang, 128 Academia Road, Sec. 2, Taipei 115, Taiwan.

[2] Taiwan International Graduate Program (TIGP), Sustainable Chemical Science and Technology (SCST), Academia Sinica, Taipei 115, Taiwan

[3] Department of Applied Chemistry, National Chiao Tung University, Hsinchu, Taiwan

[4] Institute of Chemistry, Chemistry, Academia Sinica, Nankang, 128 Academia Road, Sec.2, Taipei 115, Taiwan

* Correspondence: bruceyu@gate.sinica.edu.tw; Tel.: +886-2-55728634

Received: date; Accepted: date; Published: date

Supplementary Materials:

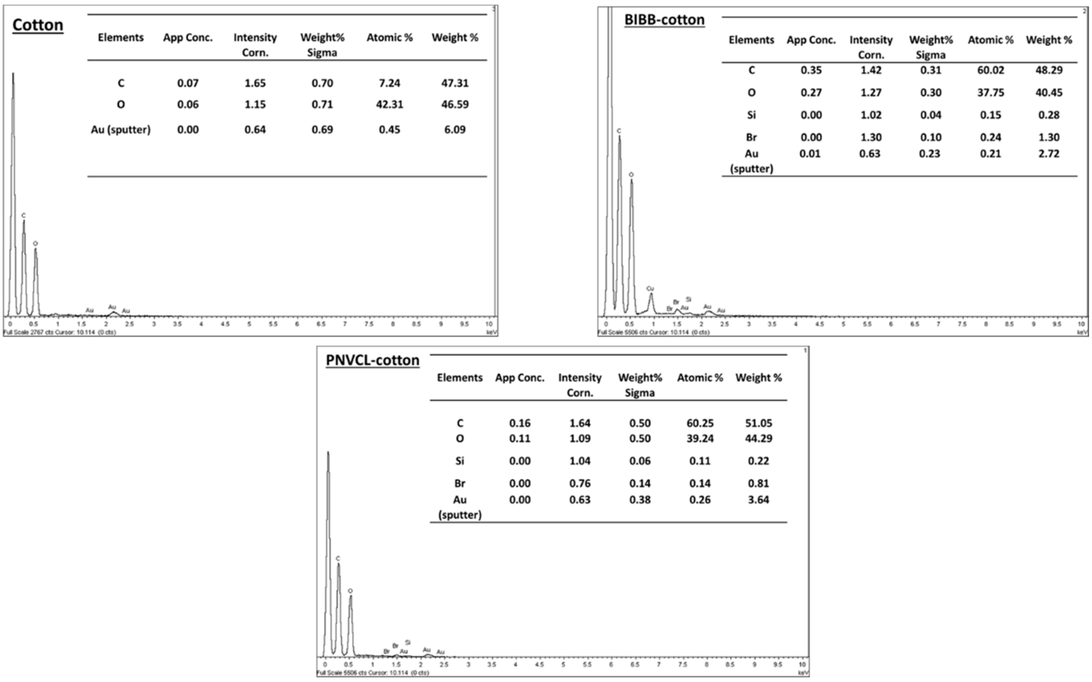

Figure S1. EDS spectra and elemental measurements of the Cotton, Cotton-BiBB and Cotton-PNVCL fabrics.

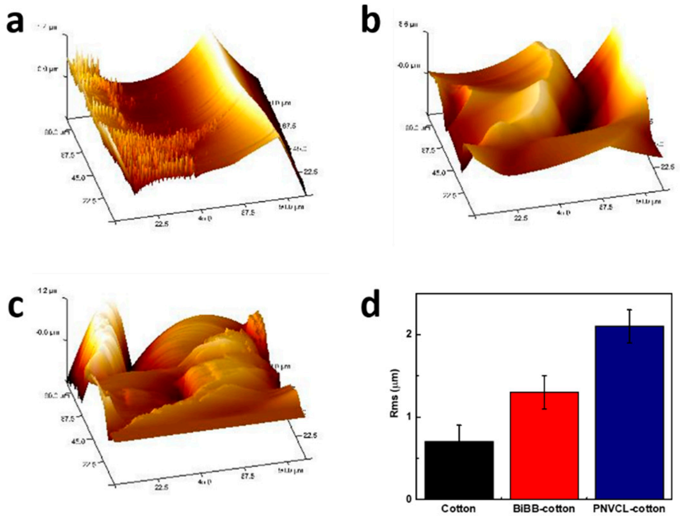

Figure S2. (a–c) AFM images of the (a) Cotton, (b) Cotton-BiBB, and (c) Cotton-PNVCL fabrics. (d) Quantitative roughness analysis of each of the fabrics.

Polymerization time (H)	Before polymerization (Wa)	After polymerization (Wb)	Amount of polymer grafted (Wb-Wa)	Grafting yield (%)	Average	Standard deviation
2	0.3	0.33	0.03	9.1	9.5	1.2
	0.32	0.35	0.03	8.6		
	0.33	0.37	0.04	10.8		
4	0.27	0.31	0.04	12.9	12.0	1.5
	0.35	0.39	0.04	10.3		
	0.34	0.39	0.05	12.8		
6	0.35	0.42	0.07	16.7	17.0	1.0
	0.36	0.44	0.08	18.2		
	0.36	0.43	0.07	16.3		
8	0.34	0.45	0.11	24.4	25.2	0.9
	0.3	0.4	0.1	25.0		
	0.31	0.42	0.11	26.2		
10	0.29	0.43	0.14	32.6	32.6	0.7
	0.32	0.47	0.15	31.9		
	0.3	0.45	0.15	33.3		
12	0.25	0.41	0.16	39.0	37.1	3.8
	0.32	0.53	0.21	39.6		
	0.35	0.52	0.17	32.7		
14	0.32	0.53	0.21	39.6	37.8	3.9
	0.34	0.57	0.23	40.4		
	0.36	0.54	0.18	33.3		
16	0.36	0.62	0.26	41.9	39.1	3.8
	0.35	0.59	0.24	40.7		
	0.3	0.46	0.16	34.8		

Figure S3. Quantitative data for grafting yield analysis used to plot figure 2a.

© 2020 by the authors. Submitted for possible open access publication under the terms and conditions of the Creative Commons Attribution (CC BY) license (http://creativecommons.org/licenses/by/4.0/).
